# Time Trends Analysis of Cervical Cancer Incidence in Cluj County, Romania, Using Data from a Population-Based Cancer Registry

**DOI:** 10.3390/curroncol28030159

**Published:** 2021-04-30

**Authors:** Radu-Mihai Ignat, Daniela Coza, Patricia Ignat, Radu-Ion Badea, Ofelia Șuteu

**Affiliations:** 1 Faculty of Medicine, “Iuliu Haţieganu” University of Medicine and Pharmacy, 400012 Cluj-Napoca, Romania; ignat.radu@umfcluj.ro (R.-M.I.); rbadea@umfcluj.ro (R.-I.B.); ofelia.suteu@iocn.ro (O.Ș.); 2 Department of Cancer Prevention and Control, “Prof. Dr. Ion Chiricuţă” Oncology Institute, 400015 Cluj-Napoca, Romania; dana.coza@iocn.ro; 3 Department of Radiotherapy, “Prof. Dr. Ion Chiricuţă” Oncology Institute, 400015 Cluj-Napoca, Romania;

**Keywords:** cervical cancer, incidence time trends, data quality, organized screening program, population-based cancer registry

## Abstract

(1) Background: Romania has one of the highest cervical cancer incidence rates in Europe. In Cluj County, the first screening program was initiated in 1998. We aimed to investigate the time trends of cervical cancer incidence in women from Cluj County and to evaluate the data quality at the Cancer Registry. (2) Methods: We calculated time trends of standardized incidence rates in the period 1998–2014 and the Annual Percent Change (APC%). To assess data quality, we used the indicators: mortality/incidence ratio (M/I), percentage of cases declared only at death (DOD%), and percentage of cases with pathological confirmation (PC%). (3) Results: The standardized incidence rate increased steadily, from 23.74 cases/100,000 in 1998, to 32/100,000 in 2014, with an APC% of 2.49% (*p* < 0.05). The rise in incidence affected both squamous cell carcinoma (APC% 2.49%) (*p* < 0.05) and cervical adenocarcinoma (APC% 10.54%) (*p* < 0.05). The M/I ratio was 0.29, DOD% 2.66%, and MC% 94.8%. The last two parameters are within the silver standard concerning data quality. (4) Conclusions. Our study revealed an ascending trend of cervical cancer incidence, more consistent for adenocarcinoma, in the context of a newly introduced screening program and partially due to the improvement of the quality of case reporting at the Cancer Registry from Cluj.

## 1. Introduction

In the last two decades, Romania constantly recorded the highest incidence and mortality rates of cervical cancer in Europe [1,2,3], ranking 5th in Europe in 2018, according to estimates in the GLOBOCAN Project in the context of a marked downward trend in cervical cancer incidence and mortality worldwide, in countries that have adopted national screening programs [4,5].

The unfavorable situation in our country regarding the incidence and mortality of this highly preventable cancer reflects the inefficiency of previous actions and measures of secondary cancer prevention. These consisted exclusively of opportunistic screening, performed in recent decades, with insufficient coverage of the target female population according to unanimously accepted international standards [1,6,7].

Cervical cancer is an important public health problem, due to the premature mortality it causes, the deterioration of the quality of life, and the substantial resources that must be allocated for diagnosis and treatment. Assessing the burden, estimating and monitoring time trends of the disease are essential for planning medical resources and evaluating both the economic impact of the disease and the efficiency of primary and secondary prevention measures [8]. Cervical cancer trends mainly depend on the existence of effective screening programs, in conjunction with an HPV vaccination strategy. In the next decades, screening combined with vaccination are expected to eliminate cervical cancer in low- and middle-income countries, which hold the greatest burden of this cancer [9,10,11,12].

We performed a retrospective analysis of cervical cancer cases in Cluj County, using data from the North-Western Cancer Registry, to assess whether the trends of cervical cancer incidence and mortality were influenced by the introduction of the organized screening program by cyto-vaginal smear in our county. We aimed to investigate the trends of cervical cancer incidence in Cluj County, in the period 1998–2014 and to evaluate the data quality at the cancer registry, for an objective assessment of cervical cancer incidence trends.

## 2. Materials and Methods

We performed a descriptive study, based on data from the Territorial Cancer Registry of the North-Western Region of Romania, located in the “Prof. Dr. Ion Chiricuță” Oncology Institute, Cluj-Napoca [13].

### 2.1. Study Participants

Cervical cancer cases were selected by C53 topographic codes, according to the International Classification of Diseases for Oncology, Third Edition [14].

We included all patients of all ages with permanent residence in Cluj County, diagnosed with cervical cancers in the period 1998–2014, recorded in the Territorial Cancer Registry. The pathologic subtypes included were squamous cell carcinoma (pathological code 8070–8120), adenocarcinoma (code 8140–8490), and other pathologic types (8020, 8098, 8200, and 8560, including unconfirmed anatomopathological cases).

Patients with lymphomas (codes 9670–9680), sarcomas, mixed epithelial and mesenchymal tumors (codes 8805–9540), and neuroendocrine carcinomas (code: 8013, 8041, 8240, and 8249) of the cervix were excluded from the analysis.

After applying these criteria, we selected 2563 cases, of which 1519 were diagnosed in the period 2006–2014.

### 2.2. Data Collection

We collected data from the Territorial Cancer Registry of the North-Western Region of Romania regarding age, date of diagnosis, site, and anatomopathological type of the tumor.

For the interval 2006–2014, information was available for the following parameters: stage at diagnosis, according to the TNM classification [15], and date of death.

Demographic data of the female population in Cluj County, in the period 1998–2014, by 5-year age groups was obtained from the National Institute of Statistics [16].

### 2.3. Statistical Analysis

We calculated the age-standardized incidence rate per 100,000 population by the direct method, using the standard world population, firstly for all cases, then by pathological types and by stages for the period 2006–2014 [17]. The age-specific incidence rate was calculated for the period 1998–2014.

Time trends of incidences rates were calculated using the Joinpoint Regression program, version 4.1.1, Bethesda, USA [18]. We used the log-linear regression model. We identified time change points, and we estimated the Annual Percent Change (APC%), assuming that the rates change with a constant percentage on a logarithmic scale for each time segment [19].

The quality control of the data regarding cervical cancer cases registered at the Territorial Cancer Registry in the period 2006–2014 was performed to assess the completeness of the data collected at the registry. This analysis was performed using the following indicators: mortality/incidence ratio (M/I), using crude rates, percentage of cases declared only at death (DOD%), and percentage of cases with pathological confirmation (PC%) [13].

The statistical program used was SPSS 17. To compare the variables in different subgroups, the χ^2^ test was applied. In the statistical analysis, all tests used were considered statistically significant at *p* < 0.05.

## 3. Results

In the period 1998–2014, a number of 2563 new cases and 868 deaths were registered. The number of new cases, deaths, and crude and standardized incidence and mortality rates are shown in Table 1. The average age at diagnosis was 50.2 years (range 18–99 years; standard deviation ± 14). The majority (84.7%) were squamous cell carcinomas, 7.7% were adenocarcinomas, and 7.7% were other types and unconfirmed anatomopathological cases.

**Figure 1:**
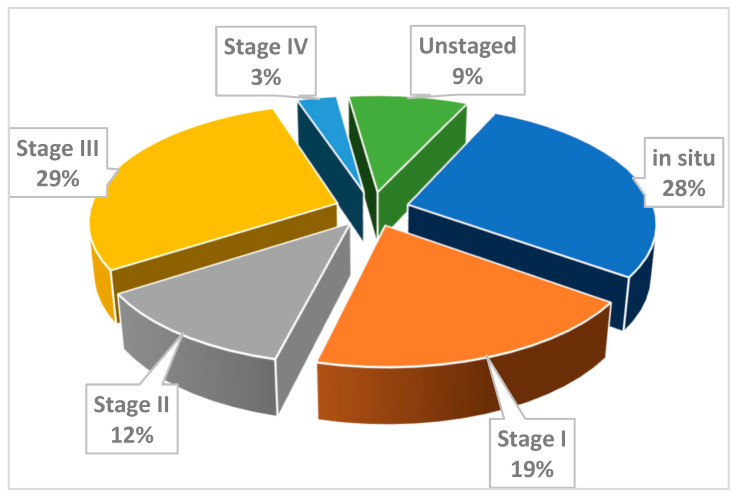
Distribution of new cases of cervical cancer by stages, Cluj County, 2006–2014.

The distribution of new cases by stages is presented in Figure 1. A high proportion of in situ and stage I cases was observed in the period 2006–2014.

**Figure 2:**
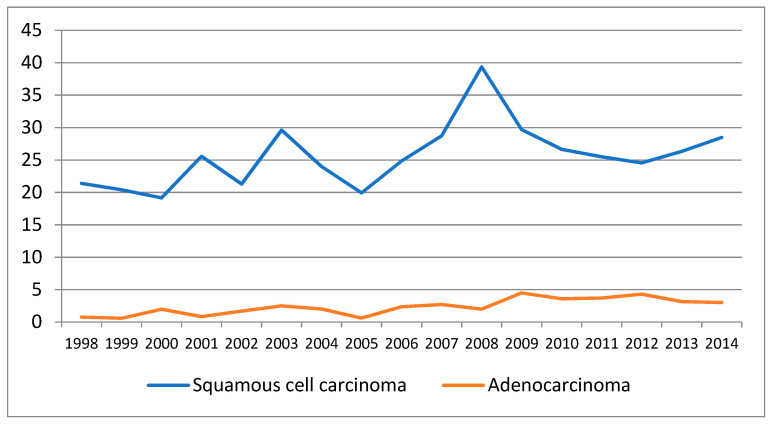
Evolution of standardized incidence rates (SIR) of squamous cell carcinoma and adenocarcinoma, Cluj County, 1998–2014.

The standardized incidence rate for both pathological types increased steadily, from 23.74/100,000 in 1998 to a maximum of 46/100,000 in 2008 and reached 32 cases per 100,000 in 2014 (Table 1 and Figure 2).

**Figure 3:**
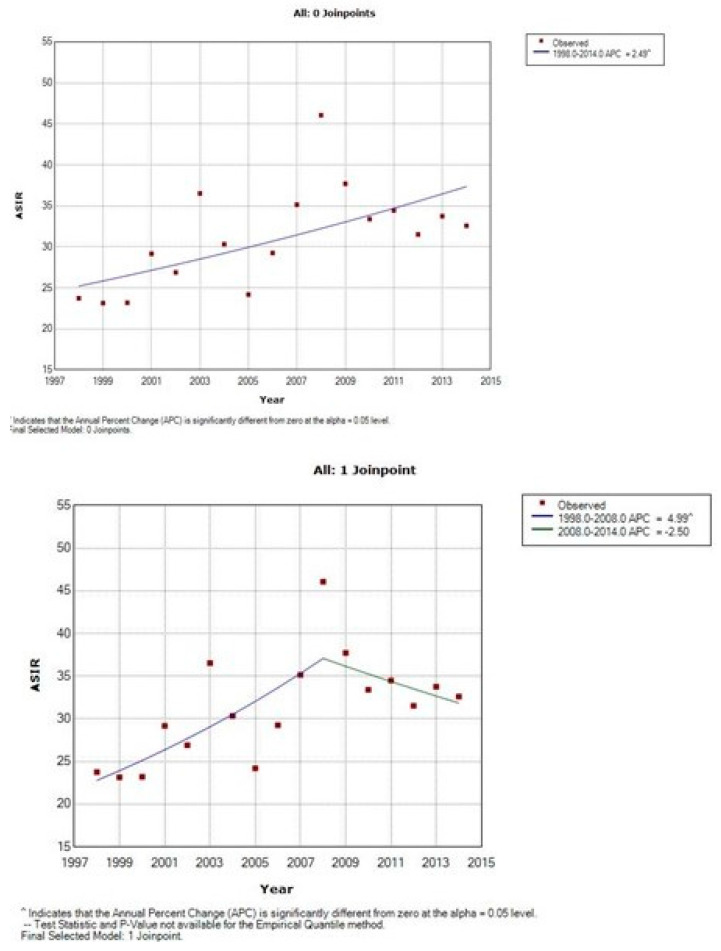
Time trends of age-standardized incidence rates of cervical cancer, Cluj County, 1998–2014.

**Figure 4:**
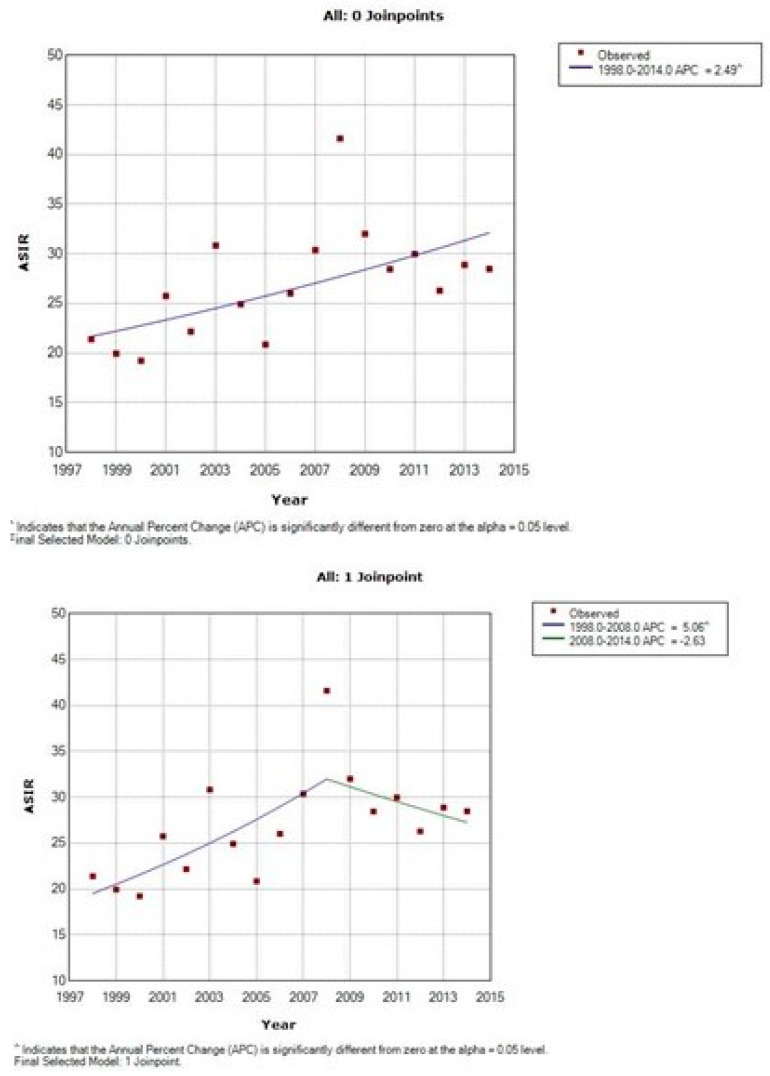
Time trends of age-standardized incidence rates of squamous cell carcinoma of cervix uteri, Cluj County, 1998–2014.

**Figure 5:**
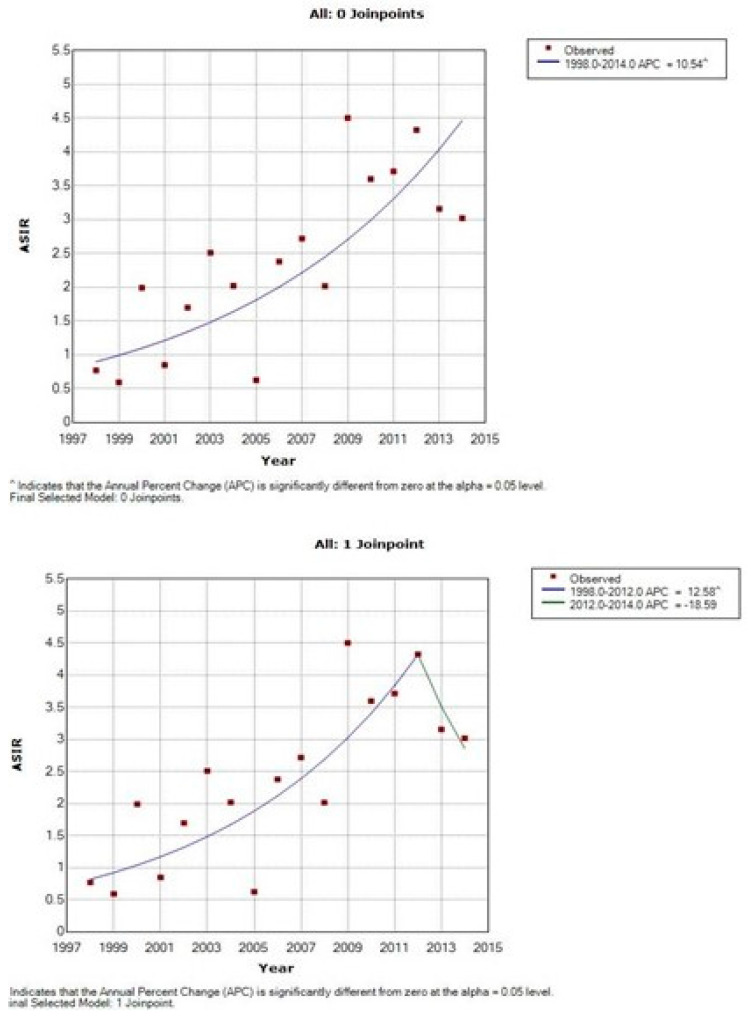
Time trends of age-standardized incidence rates of adenocarcinoma of cervix uteri, Cluj County, 1998–2014.

The time trends of the incidence are presented in Table 2, Table 3 and Table 4 and Figure 3, Figure 4 and Figure 5. There is an overall statistically significant increase in the studied period, of 2.49%, with a more pronounced rise, of 4.99% in the period 1998–2008. Adenocarcinoma increased considerably, with 10.54% per year between 1998 and 2014 and 12.58% per year in the period 1998–2012 (Figure 5 and Table 2).

Concerning the age-specific incidence rates, the highest increase was registered in the age group 25–34 years (APC = 12.36%) (*p* = 0.01) between 1998 and 2009, immediately after the introduction of the screening program, due to the marked increase in squamous cell carcinoma (APC = 12.71%), and for the age groups 55–64 between 2000 and 2014, with an APC = 5.83% (*p* = 0.01) (Table 3 and Table 4). For women aged 45 years and over, a very important rise was registered for adenocarcinoma (Table 4).

The evolution of the data quality indicators at the Cancer Registry in the period 2006–2014 showed that the M/I ratio had an average value of 0.29 (Table 5). The DOD% decreased from 2.84 in 2006 to 1.23 in 2014, reflecting an improvement in case reporting to the Territorial Cancer Registry. The PC% of cases remained high, around 95%.

## 4. Discussion

This is the first population-based study to analyze detailed trends of incidence rates of cervical cancer in Cluj County based on observed data. Our study used routinely collected and published data at the Territorial Cancer Registry from the North-Western Region of Romania on cervical cancer cases diagnosed in Cluj County. Between the 17 years considered, there is a statistically significant increase in the incidence for both pathological types, with a predominant involvement of women of working age. The increase was more pronounced for adenocarcinoma as well as for women aged over 45 years. The high quality of the data at the Territorial Cancer Registry proves the exhaustiveness of the information about newly diagnosed cases in Cluj County, allowing a correct analysis of the incidence time trends.

In most industrialized countries, both incidence and mortality from cervical cancer have decreased in recent decades, this considerable reduction being largely attributed to the introduction of screening programs, by conventional cytology or HPV testing in the past years [4,5,20,21,22]. This favorable trend has recently been reversed in some countries, where the incidence and mortality are rising among young women, more likely due to changes in sexual behavior, increased prevalence of smoking, and use of oral contraceptives, but also insufficient coverage in screening programs [23,24].

In 1998, in Cluj County, a pilot screening program for cervical cancer was implemented, by conventional cyto-vaginal smear, targeting women aged 25–64 [1]. From 2002, the Ministry of Health (M of H) financed the performance of cyto-vaginal tests in several counties of the country, including Cluj County [1,25,26,27,28,29]. In 2012, the screening program expanded nationwide, according to the European Guidelines for quality assurance of screening programs [7,30]. The program was funded by the M of H, targeting women aged 25–64 years, who were tested every 5 years.

The link between the screening program and the Cancer Registry is mandatory in ensuring the medium- and long-term evaluation of the screening program [8]. In Romania, cancer reporting is compulsory since the issue of an Order of the M of H in 1980, updated in 2002 and 2007.

In Cluj County, in the period 1998–2014, the incidence rates of cervical cancer increased steadily. The increasing trends for squamous cell carcinoma between 1998 and 2008 and adenocarcinoma between 1998 and 2012, together with a high proportion (28%) of cases of in situ and stage I carcinoma diagnosed between 2006 and 2014 are explained by the onset of the pilot screening program in 1998 in Cluj County, followed by its expansion in 2002, when a more significant number of cases was identified [1]. This increase is followed in 2008 by a sudden drop to values recorded in the first years of the county-organized program, an effect due to the initial identification of both incident and prevalent cases in the first years of screening, followed by the subsequent identification of only incident cases in the program [13].

Whether this rise in incidence is real or apparent is up to debate. The hypothesis of a real increase in incidence is supported by the significant increase in both pathological types especially in the age groups 25–34 and 55–64 years old, suggesting a better compliance and participation of these age groups in the screening program. The substantial rise of the incidence in the age group 55–64 years is in accordance with the results obtained in a previous study performed in our county using HPV testing, which highlighted a second peak of incidence in postmenopausal women, possibly attributed to population and viral characteristics, age-related anatomopathological changes, as well as to a latent HPV infection, with later reactivation [29,31,32]. This aspect was identified in some developed countries, Eastern Europe, and in many low- and middle-income countries worldwide [33,34]. The rise of the incidence in younger age-groups is consistent with data cited in the literature and is explained by changes in sexual behavior [24], whereas for the age group 55–64 by insufficient coverage in the opportunistic screening performed before 2002, which was not capable to reduce the incidence [35,36].

To an extent difficult to assess based on this study, the apparent increase in incidence can be attributed to improved diagnostic tools, but also to a more accurate registration of cases due to legislative regulations on mandatory cancer reporting. For Cluj County, we consider that the more frequent detection of cases by screening explains, at least in part, the abrupt increase in incidence during 1998–2008, hypothesis supported by the increase in specific incidence in the age groups eligible for screening, 25–65 years, as well as the high percentage of cases diagnosed in early stages. This aspect correlates with the stationary evolution of mortality, through diagnosis and early treatment of “in situ” cases, within the screening program. The very high increase in APC%, especially for adenocarcinoma, in a very short period, can also be explained by improvements in cancer surveillance coverage.

In the European context, our results show very high incidence rates, compared even with less developed countries, based on observed data published in Cancer Incidence in 5 Continents, in a similar retrospective period, for the populations that were included in this publication [37]. Among countries with the highest values are Estonia (19.2/100,000) and Bulgaria (18.9/100,000) in 2012. In developed countries, the incidence range is 9.8, 9.7, 8.4, and 3.7/100,000 in Denmark, Norway, Germany, and Switzerland, respectively. This important discrepancy with our data is explained by cumulating factors such as the identification of prevalent cases in a new screening program and better declaration of cases to the cancer registry, but we must take in consideration also a real increase due to the lack of an organized screening program in the past and exposure to high-risk HPV types.

In order to assess the time trends of the disease, an analysis of the validity of the data is required [38]. The completeness and quality of data from the Territorial Cancer Registry were assessed through major indicators of cancer quality proposed for this purpose. A high proportion of DOD% cases suggest that the routine data collection system does not adequately cover all healthcare facilities in the territory, including pathological anatomy laboratories. Some European and American cancer registries reach a completion rate close to 100%, with a very low proportion of DOD, and the percentage of histologically confirmed cases exceeds 95% [39].

In terms of quality of data, we found that the high data quality criteria requested for certification of cancer registries in some countries are met for our data [40]. The evaluation indicator DOD% reached the level of "silver standard," for each diagnosis year at the time of reporting. This reveals a high degree of completeness for new cervical cancer cases included in the Territorial Registry, with a value of 2.88% in the period 2006–2014, interval for which there was information regarding the deaths of existing cancer cases in the Territorial Registry [41]. The M/I ratio, as an essential indicator of the degree of completeness, given that mortality data come from vital statistics, is an independent source of data. It has been permanently sub-unitary and has been steadily declining, advocating for almost full inclusion of new cases in Cluj County, in the period 2006–2014. The percentage of pathological confirmations of almost 95% in the last half of the studied interval allowed the assessment of the validity of diagnostic information and stood witness of the high degree of recovery of information regarding newly diagnosed cases [13]. Our data provided a reasonably accurate portrayal of the cervical cancer time trends especially in the last years (2006–2014) since improvements in cancer surveillance coverage occurred based on legislative changes [13].

The strengths of the study rely on the global relevance of our findings, since the study was conducted on the population of Cluj County, for 17 years. This is the first study in Romania to assess the time trends of cervical cancer incidence after the introduction of the organized screening program. No data exist in the literature showing detailed time trends of cervical cancer incidence in Romania, with respect to age distribution, pathologic subtype, and stage. A major strength of our study is that we relied on observed data, that avoids the uncertainty of using estimated data and allowed us to accurately portray the cervical cancer burden in our county. This is also the first published study in Romania that assessed the data quality of a Cancer Registry. The completeness of data at the Territorial Registry of Cancer is high, supported by the silver standard of the parameters used in the evaluation. The high quality of the data proves the exhaustiveness of the information on newly diagnosed cases in Cluj County, allowing a correct analysis of incidence trends.

There are some limitations of this study: the current analysis was on inhomogeneous data quality, with constant improvement of the quality during the studied interval. Since it takes several years for a registry to collect, process, check, and validate the quality of data in an accurate manner and report sufficiently reliable and complete cancer data for a given year, we presented delayed data, for the year 2014. Data about staging was only available since 2006, and about the quality of data parameters since 2008, due to the reorganization of activities at the Cancer Registry in 2008. The comparisons of our results with data at national level are difficult because those are only estimated data from GLOBOCAN 12 and 18 [3,5] and we pointed out that our regional data are observed.

## 5. Conclusions

Based on the high quality of the data provided by the Cancer Registry, our study revealed a very high incidence of cervical cancer in Cluj county. The ascending trend of cervical cancer incidence, more consistent for adenocarcinoma, can be explained in the context of a newly introduced screening program. The first beneficial effect in the medium term of the organized screening program is represented by the high percentage of cases detected in early stages. To further influence the incidence and mortality of cervical cancer, screening must continue, with quality control of the program at all stages. Improving and maintaining the high quality of the data collected at the Territorial Cancer Registry is a mandatory condition to fulfill their mission of accurately reflecting the cancer burden and ensuring the quality control of the primary and secondary prevention programs in the future.

## Figures and Tables

**Table 1:** Annual incidence and mortality rates from cervical cancer, Cluj County, 1998–2014.

	1998	1999	2000	2001	2002	2003	2004	2005	2006	2007	2008	2009	2010	2011	2012	2013	2014	Total
**new cases**	117	118	110	136	127	170	147	119	140	159	221	183	162	172	156	163	163	2563
**CIR**	31.49	31.5	29.8	36.7	35	48.4	41.4	34.1	40.14	45.6	63.3	52.5	46	48	43.2	45.2	45.2	-
**SIR**	23.74	23.13	23.19	29.16	26.88	36.5	30.3	24.18	29.24	35.14	46.06	37.7	33.4	34.46	31.51	33.45	32	-
**Deaths**	61	60	58	48	57	45	44	57	51	51	40	52	35	47	45	58	59	868
**CMR**	16.49	16.21	16.26	14.9	19.4	19	23.4	16.3	14.62	14.62	11.46	14.9	9.96	13.3	11.4	14.21	14.2	-
**M/I**	0.52	0.51	0.53	0.35	0.45	0.26	0.30	0.48	0.36	0.32	0.18	0.28	0.22	0.27	0.29	0.36	0.36	0.34
**squamous cell carcinoma**																		
**New cases**	102	98	90	119	102	140	116	98	123	134	194	151	135	146	125	135	137	1989
**SIR**	21.39	20.41	19.16	25.57	21.3	29.62	24	19.94	24.86	28.74	39.36	29.7	26.65	25.5	24.55	26.33	28.48	-
**adenocarcinoma**																		
**New cases**	4	3	10	4	9	11	10	4	12	13	11	23	19	21	25	17	17	196
**SIR**	0.77	0.59	1.99	0.85	1.7	2.51	2.02	0.62	2.38	2.72	2.01	4.5	3.6	3.71	4.32	3.16	3.02	-
**other PATHOLOGICAL types (including 8000/3)**																		
**New cases**	11	17	10	13	16	19	21	17	6	12	16	9	9	6	6	9	9	197

CIR = crude incidence rate; SIR = standardized incidence rate; CMR = crude mortality rate; M/I = mortality/incidence ratio.

**Table 2:** Time trends of age-standardized incidence rates (ASIR) (cases/100,000) of cervical cancer, Cluj County, 1998–2014.

Pathologic Subtypes	1998 ASIR (No)	2014 ASIR (No)	Joinpoint Analysis (1998–2014)					
			Trend 1		Trend 2		Trend 3	
			Period	APC%	Period	APC%	Period	APC%
Cervix uteri (all)	23.74 (117)	32.58 (163)	1998–2014	2.49 *	1998–2008	4.99 *	2008–2014	−2.5
Squamous cell carcinoma	21.4 (102)	28.5 (137)	1998–2014	2.49 *	1998–2008	5.06 *	2008–2014	−2.63
Adenocarcinoma	0.77 (4)	3.02 (17)	1998–2014	10.54 *	1998-2012	12.58 *	2002–2014	−18.59

* *p* < 0.05.

**Table 3:** Time trends of the age-specific incidence rates (ASpIR) (cases/100,000), 1998–2014, and of the age-standardized incidence rates (ASIR) (cases/100,000) by stage, 2006–2014, Cluj County.

Cervix Uteri by Age Group	1998 ASpIR (No)	2014 ASpIR (No)	Joinpoint Analysis (1998–2014)					
			Trend 1		Trend 2		Trend 3	
			Period	APC%	Period	APC%	Period	APC%
• 25–34	25.84 (14)	28.45 (17)	1998–2014	4.74 *	1998–2009	12.36 *	2009–2014	−15.62
• 35–44	60.85 (32)	81.49 (42)	1998–2014	1.34	1998–2001	9.99	2001–2014	0.34
• 45–54	55.98 (26)	65.25 (28)	1998–2014	0.83	1998–2001	17.61	2001–2014	−1.03
• 55–64	48.84 (21)	78.36 (38)	1998–2014	5.05 *	1998–2000	−7.24	2000–2014	5.83 *
• ≥65	44.11 (24)	52.94 (36)	1998–2014	1.17	1998–2000	−16.39	2000–2014	2.33
**Cervix Uteri by Stage**	**2006** **ASIR (No)**	**2014** **ASIR (No)**	**Joinpoint Analysis (2006–2014)**					
			**Trend 1**		**Trend 2**		**Trend 3**	
			**Period**	**APC%**	**Period**	**APC%**	**Period**	**APC%**
• 0 “in situ”	7.65 (32)	9 (41)	2006–2014	−3.98	2006–2008	35.16	2008–2014	−11.07
• I	4.67 (21)	6.36 (28)	2006–2014	−0.93	2006–2008	36.44	2008–2014	−7.79
• II	4.42 (20)	5.8 (29)	2006–2014	3.84	2006–2008	–15.87	2008–2014	8.86 *
• III	9.56 (48)	8.84 (48)	2006–2014	−1.33	2006–2011	1.45	2011–2014	−6.65
• IV	0.3 (2)	0.39 (2)	2006–2014	−5.93	2006–2008	40.24	2008–2014	−14

* *p* < 0.05.

**Table 4:** Time trends of the age-specific incidence rates (ASpIR) (cases/100,000), by pathological type, 1998–2014.

Cervix Uteri by Age Group	1998 ASpIR (No)	2014 ASpIR (No)	Joinpoint Analysis (1998–2014)					
			Trend 1		Trend 2		Trend 3	
			Period	APC%	Period	APC%	Period	APC%
Squamous cell carcinoma								
• 25–34	25.84 (14)	26.78 (16)	1998–2014	4.72	1998–2009	12.71 *	2009–2014	−16.51
• 35–44	53.24 (28)	75.67 (39)	1998–2014	1.65	1998–2008	3.60	2008–2014	−2.27
• 45–54	55,98 (26)	58.26 (25)	1998–2014	0,39	1998–2001	11.83	2001–2014	−0.91
• 55–64	41.86 (18)	66 (32)	1998–2014	4.95 *	1998–2000	–11.47	2000–2014	6.03 *
• >64	29.4 (16)	33.82 (23)	1998–2014	1.02	1998–2001	–12.30	2001–2014	2.76
Adenocarcinoma								
• 25–34	0 (0)	1.67 (1)	2004–2014	–2.55	2004–2009	11.27	2009–2014	−13.07
• 35–44	3.80(2)	5.82(3)	1998–2014	4.40	1998–2000	27.60	2000–2014	3.14
• 45–54	0 (0)	7 (3)	1999–2014	9.93 *	1999–2012	12.44 *	2012–2014	−21.30
• 55–64	2.32 (1)	8.25(4)	1998–2014	6.49 *	1998–2002	16.26	2002–2014	4.79
• >64	1.83 (1)	8.82 (6)	1998–2014	11.13 *	1998–2012	12.47 *	2012–2014	−9.12

* *p* < 0.05.

**Table 5:** Quality control of data on cervical cancer cases registered at the Territorial Cancer Registry of the North-Western Region of Romania, period 1998–2014.

Indicator	1998	2006	2007	2008	2009	2010	2011	2012	2013	2014	Total 2006–2014
M/I	0.52	0.36	0.32	0.18	0.28	0.22	0.27	0.29	0.36	0.36	0.29
DOD%	–	2.84	3.16	3.64	3.31	3.11	1.89	2.55	1.86	1.23	2.66
PC%	–	97.16	92.41	93.18	95.03	94.41	96.86	96.18	95.03	94.48	94.87

## Data Availability

Data are unavailable due to patient confidentiality.
